# An Evaluation of Variations in the Carpal Tunnel Dimensions of Adult Subjects in a Hospital-Based Population: An Ultrasonographic Cross-Sectional Study

**DOI:** 10.7759/cureus.56001

**Published:** 2024-03-12

**Authors:** Mamta Rani, Nisha Yadav, Monika Srivastava, Anuj Jain, Nityanand Srivastava, Anurag Yadav, Vishal R Jasuja

**Affiliations:** 1 Anatomy, Uttar Pradesh University of Medical Sciences, Saifai, IND; 2 Physiology, Uttar Pradesh University of Medical Sciences, Saifai, IND

**Keywords:** carpal tunnel syndrome, transducers, compression, scaphoid, trapezium, pisiform, hook of hamate, anatomy at the wrist, body mass index, adult

## Abstract

Background

The carpal tunnel is a groove that spans the palm as a ‘U.’ The ulnar and radial sides of the wrist are made up of the scaphoid tubercle and trapezium while the palmar aspect is made up of carpal bones. Our study aimed to see whether there were differences in carpal tunnel size between men and women.

Material and methods

The study was conducted on 65 healthy adults, 13 (20%) were males and 52 (80%) were females (both non-pregnant and pregnant). Inclusion criteria were healthy adults and bilaterally symmetrical limbs. Exclusion criteria were chronic disease, diabetes, hypertension, immunological disorders, any visible abnormalities, and a history of upper extremity pain on either side. A high-resolution ultrasound machine with a linear transducer was used to perform an ultrasound scan of the carpal tunnel. The anteroposterior dimension was measured at the midline, or along the axis of the middle finger, and the transverse diameter was measured at the midpoint of the flexor retinaculum. The cross-sectional area of the tunnel was measured at its largest diameter within the carpal tunnel. All the dimensions were measured in centimeters.

Results

The mean transverse diameter of the right side was 1.824 ± 0.223 cm (p-value 0.002) and of the left side was 1.742 ± 0.197 cm (p-value 0.004). The mean cross-sectional area of the carpal tunnel on the right side was 1.417 ± 0.379 cm^2^ (p-value 0.008) and on the left side was 1.306 ± 0.303 cm^2^ (p-value 0.004), respectively. Age, sex, weight, and BMI were discussed. The carpal tunnels of females were found to be comparatively squarer and smaller than those of males.

Conclusion

The transverse diameter and cross-sectional area of the carpal tunnel and their correlation with carpal tunnel syndrome are predicted by age, sex, weight, and BMI. Both sexes had the same wrist ratio.

## Introduction

Anatomically the carpal tunnel (CT) is a ‘U’-shaped groove that runs through the palm. It is made up of the carpal bones at the palmar aspect of the wrist. It comprises the scaphoid tubercle on the ulnar side and trapezium on the radial side.

On ultrasound (US), it is seen as an oval space with the carpal ligaments on the top and the flexor retinaculum on the bottom, appearing as a well-defined space with round punctate hyperechoic structures defining the tendons and nerve if seen in the short axis of the wrist. The pisiform and the hook of the hamate in the middle and the inside of the round connective tissue extend from the flexor retinaculum to the scaphoid and trapezium on both sides. This definition includes the median nerve and the eight tendons from the flexor digitorum superficialis and flexor digitorum profundus, along with a single tendon of the flexor pollicis longus [1].

Carpal tunnel syndrome (CTS) is a painful condition of the wrist, which develops due to the median nerve, in its course within the carpal tunnel, getting compressed due to any cause. This increase in pressure is augmented by the passive extension of the digits; as the fingers are flexed, the extensor tendons on the dorsal aspect help release pressure by relaxing [2].

Motor or sensory deficits in the median nerve's distribution, such as pain or numbness over the hand's lateral palm and the first two fingers, as well as weakness or paralysis of the thenar muscles, define CTS [3].

Most studies suggest that a higher incidence of CTS is seen in women as compared with men [4-9]. Several studies taking this into account have been conducted by comparing height, body mass index (BMI), occupational differences, educational attributes, and age [7-9]. Some studies have tried to explain the onset of CTS by comparing the anatomical variations in hands and wrists [8-9].

Some studies have suggested that people who have wrists with the palmar-dorsal dimension ratio to the radioulnar dimension, or between depth to width, approaching 1.0, are more likely to develop CTS compared to others [10-11].

Moghtaderi et al. observed in a study that women in the group with CTS also had higher BMI and squarer wrists than women in the control group. However, there were also differences within their control group, in which men with no CTS had lower BMI and fewer square wrists when compared with women in this group. This could not be compared to the group with CTS, as their study had a limited number of men with CTS [11].

Boz et al. found in a study that wrists that were ‘squarer’ were linked to CTS in women more than in men. They also observed that women in their study were shorter and had smaller hands than men, but they did not find any significant difference in the shape of the wrist between men and women [12].

Kamolz et al. conducted a study comparing 50 patients with CTS to 50 healthy controls. The results indicated that CTS was associated with shorter hands, deeper palms, and squarer wrists. However, the study did not investigate differences between the sexes in hand, wrist, and carpal tunnel dimensions and shapes [13].

Our study aimed to find out whether the size of the carpal tunnel differs between men and women.

## Materials and methods

The study was conducted over the period of one month in the Department of Radiology at Uttar Pradesh University of Medical Sciences, Saifai, Etawah (India). Permission to conduct the study was granted by the institutional ethics committee (IEC No. 94/2023-24). The present cross-sectional study was conducted on healthy adults recruited for the study from the outpatient Department of Radiology. The estimated sample size was 65, based on the previous study considering an alpha error of 5% and a power of study of 80% [12]. The participants were selected randomly based on inclusion and exclusion criteria and no sexual priority was given.

The inclusion criteria were (a) healthy adults and (b) apparently bilateral symmetrical upper limbs. The exclusion criteria were (a) chronic disease, diabetes, hypertension, and immunological disorders, (b) any visible anomalies, including congenital, traumatic, or other pathologies, and (c) a history of pain in the upper limb of any side. Informed consent was obtained from the participants after explaining the purpose of the study, the procedure involved, and any potential risks or discomfort. Information was collected using a predesigned structured questionnaire under the following headings:

Demographic profile: The demographic profile of each participant was noted, e.g., age, sex, dominant hand, height, weight, and BMI.

Ultrasonographic assessment: Transverse diameter, anteroposterior diameter, and cross-sectional area measured with a high-resolution ultrasound machine with a linear transducer were used to do an ultrasound of the carpal tunnel. On an exam table, the patient sat comfortably with their arms outstretched and their palms facing upward.

**Figure 1:**
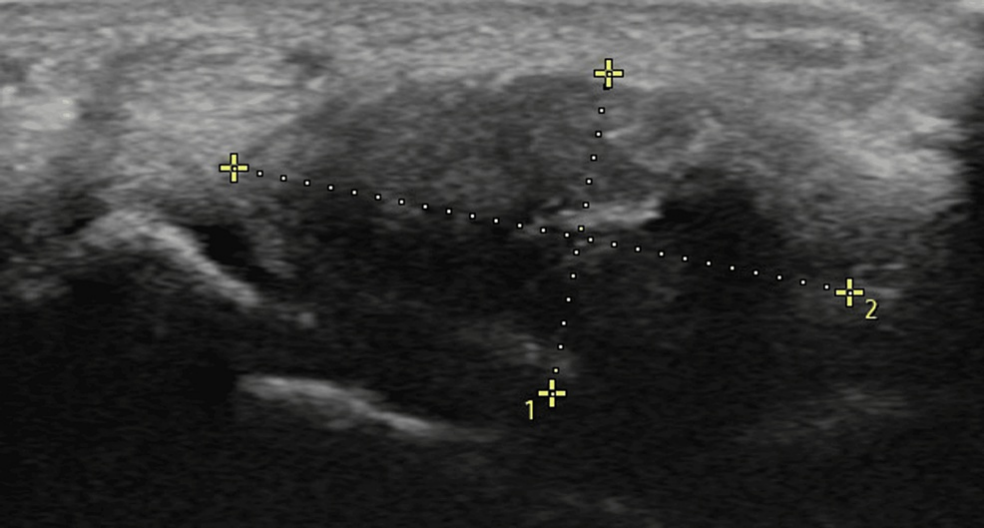
Ultrasound image of the measurement of the carpal tunnel 1. anteroposterior diameter; 2. transverse diameter

**Figure 2:**
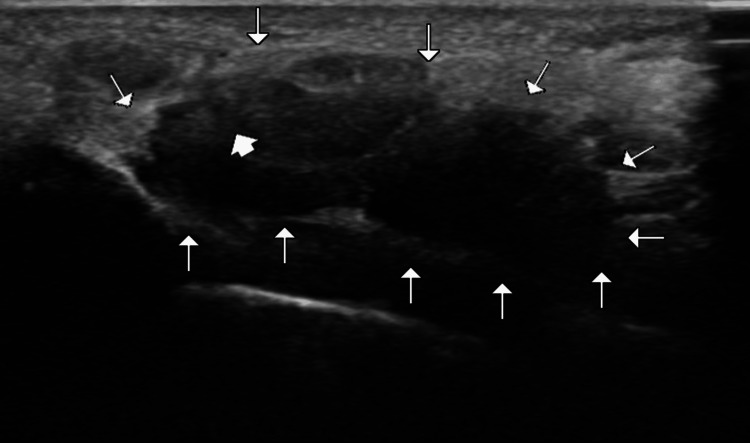
Ultrasound image of the carpal tunnel The carpal tunnel is marked by arrowheads and the median nerve is marked by a thick arrow.

The specification of the ultrasound machine was Samsung Sonoace R7, Etawah (India), Medilux Healthcare System, with a linear probe of frequency 5-15 Mhz. The transducer was placed perpendicular to the wrist crease, and the transverse view of the carpal tunnel was obtained. The ultrasound probe was placed perpendicular to the wrist crease, with the transducer marker pointing toward the radial side of the hand. The median nerve, carpal bones, flexor retinaculum, and any abnormality were identified and measured. The anteroposterior dimension was taken at the midline, or along the axis of the middle finger. The transverse diameter was taken at the midpoint of the flexor retinaculum. The tunnel was identified, and the flexor tendons were traced proximally and distally through the carpal tunnel. The cross-sectional area of the tunnel was measured at its largest diameter within the carpal tunnel (Figure 1 and Figure 2).

Statistical analysis

The data were recorded in Microsoft Excel (Microsoft Corporation, Redmond, WA, USA) and descriptive statistics of all variables were evaluated for normality and skewness. The mean, median, and standard deviations and the interquartile range were computed. The intergroup comparison was conducted by an unpaired t-test or a Mann-Whitney test based on the distribution of variables. All variables' correlations were examined with Pearson's or Spearman's correlation based on distributions.

## Results

The study was conducted on 65 subjects, which included 13 males and 52 females (29 pregnant and 23 nonpregnant). The mean age, height, and weight of subjects were 30.97 ± 14.03 years, 161.46 ± 9.76 cm, and 66.28 ± 6.7 kg, respectively. The mean duration of pregnancy was 24.7 ± 8.8 weeks. The details of descriptive statistics are mentioned in Table 1.

**Table 1:** Subject characteristics and outcome variables of carpal tunnel in normal subjects Significant difference <0.05 yr: years, cm: centimeter, RTD: right transverse diameter, RAP: right anteroposterior, RCSA: right cross-sectional area, LTD: left transverse diameter, LAP: left anteroposterior, LCSA: left cross-sectional area

Variables	Total	Male		Female	Dominant Hand	Non-dominant Hand
Sample size	65	13	52		65(R-47, L-18))	65 (L-47, R-18)
Age (yr)	30.97±14.03	40.77±20.38	28.52±10.9		NA	NA
Height (cm)	161.46±9.76	164.62±8.53	160.73±9.96		NA	NA
Weight (kg)	66.28±6.71	71.92±3.48	64.87±6.59		NA	NA
BMI (kg/m2)	25.49±2.33	26.64±1.89	25.2±2.35		NA	NA
RTD (cm)	1.824±0.223	1.985±0.185^*^	1.783±0.214^*^		1.821±0.225	1.832±0.223
RAP (cm)	0.768±0.133	0.815±0.087	0.757±0.141		0.77±0.139	0.764±0.12
RCSA (cm2)	1.417±0.379	1.625±0.283^*^	1.365±0.385^*^		1.417±0.388	1.417±0.365
LTD (cm)	1.742±0.197	1.905±0.206^*^	1.701±0.174^*^		1.756±0.221	1.737±0.19
LAP (cm)	0.744±0.117	0.794±0.08	0.732±0.122		0.735±0.087	0.748±0.127
LCSA (cm2)	1.306±0.303	1.517±0.260^*^	1.253±0.292^*^		1.301±0.287	1.308±0.312

The mean of the transverse diameter of the carpal tunnel in the right and left limbs were 1.824 ± 0.223 cm and 1.742 ± 0.197 cm, respectively. The mean anteroposterior diameter of the carpal tunnel was 0.768 ± 0.133 on the right side and 0.744 ± 0.117 cm on the left side. The cross-section area of the carpal tunnel was 1.417 ± 0.379 cm^2^ on the right side and 1.306 ± 0.303 cm^2^ on the left side.

**Figure 3:**
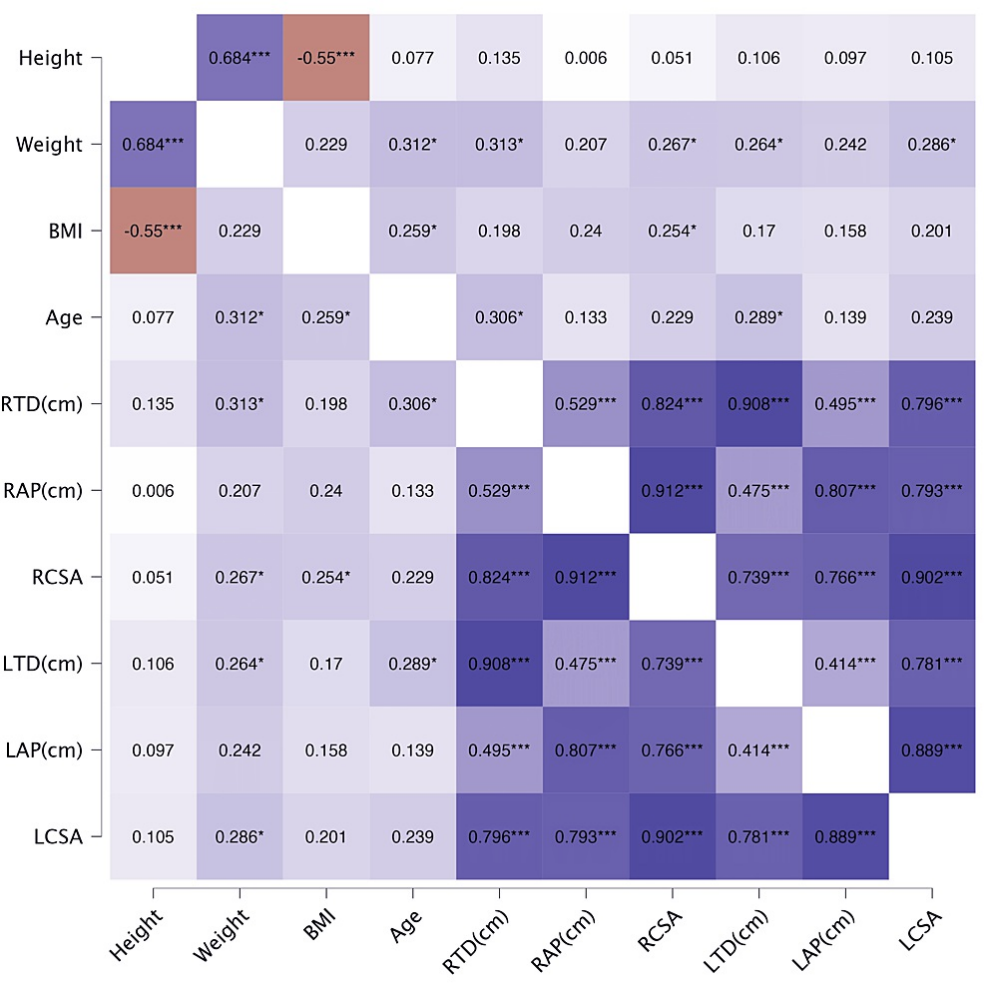
Heatmap of correlation among variables <0.05: significant, <0.01: moderate significant, <0.001: highly significant

Age, sex, weight, and BMI are predictors of the transverse diameter and the cross-section area of the carpal tunnel (Figure 3).

**Figure 4:**
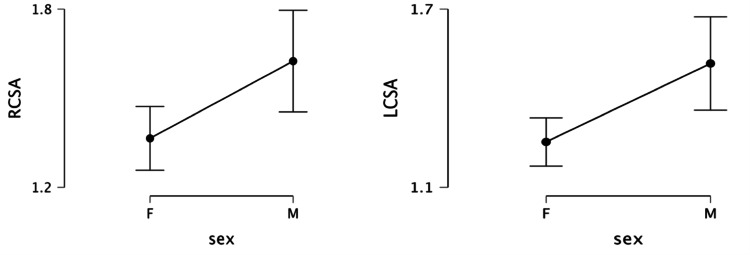
Difference in cross-section of the carpal tunnel between males and females

A significant difference in transverse diameter and cross-section area between the two sexes was observed (Figure 4).

**Figure 5:**
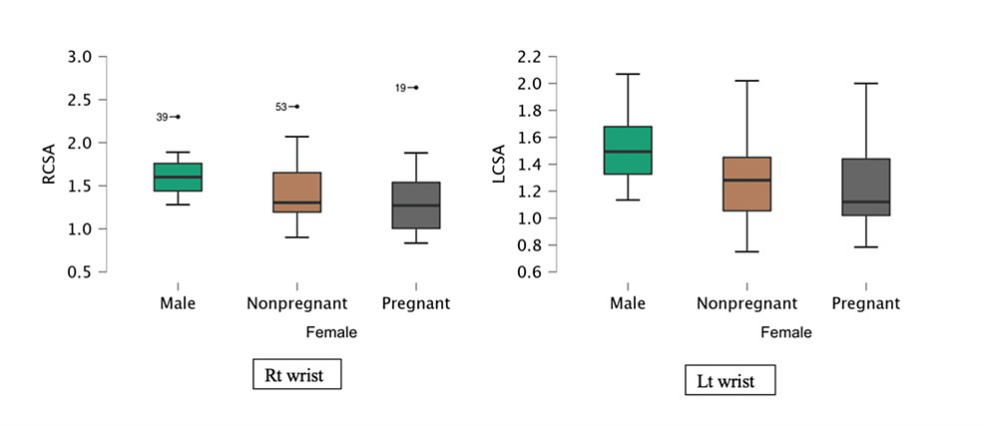
Difference of cross-sections of the right and left carpal tunnels between males and non-pregnant and pregnant females RCSA: right cross-sectional area, Rt: right, Lt: left

No significant difference was captured among women of different parity levels in relation to pregnancy because their confidence intervals were overlapping (Table 2 and Figure 5). There was no correlation observed between carpal tunnel dimension and duration of pregnancy.

**Table 2:** Mean ± SD values of variables depicted between non-pregnant and pregnant females cm: centimeter, RTD: right transverse diameter, RAP: right anteroposterior, RCSA: right cross-sectional area, LTD: left transverse diameter, LAP: left anteroposterior, LCSA: left cross-sectional area

	Non-pregnant (Mean ± SD)	Pregnant (Mean ± SD)
Sample size	23	29
Age (years)	32.17±15.19	25.62±4.16
Height (cm)	161.65±11.04	159.9±9.14
Weight (kg)	63.87±4.8	65.65±7.6
RTD (cm)	1.79±0.23	1.77±0.20
RAP (cm)	0.79±0.13	0.73±0.14
RCSA (cm2)	1.42±0.28	1.31±0.39
LTD (cm)	1.70±0.19	1.70±0.16
LAP (cm)	0.76±0.12	0.71±0.12
LCSA (cm2)	1.29±0.3	1.21±0.3

Carpal tunnels from female subjects were comparatively squarer and smaller than those from male subjects.

## Discussion

Dimension

According to Johnson et al., individuals with a wrist ratio (the ratio of wrist depth to wrist width at the level of the distal wrist crease) above 0.7 have a greater risk of developing CTS compared to those with more anteroposteriorly compressed wrists [14]. This finding has been supported by several studies, including one conducted by Moghtaderi et al. that examined 128 patients with CTS and 109 controls and confirmed that individuals with squarer wrists are more likely to develop CTS [11-18]. Our study also found that increasing age was associated with a larger cross-sectional area of the carpal tunnel.

Gender

Moghtaderi et al. reported that women with and without CTS have significantly squarer wrists than men [11]. The average wrist ratio for women (0.6981 ± 0.379) approached the threshold value reported by Johnson et al. of 0.7 [13]. However, Boz et al. did not observe this morphological difference between the wrists of different sexes in their study of Turkish participants [12]. This could be attributed to ethnic differences, as Moghtaderi et al. exclusively studied Iranian patients while Boz et al. studied Turks [11,12]. The present study did not find any significant difference in wrist ratio between males and females.

Age

Age is another predictor of carpal tunnel dimension. We did not find a correlation between age and wrist depth (anteroposterior), but our study found that increasing age was associated with a larger cross-sectional area of the carpal tunnel.

Based on these results, it seems that the larger cross-sectional area of the carpal tunnel may not be a bigger predictor of CTS in older people than the shape of the wrist. In a large study of US military members, Wolf et al. found that CTS was more common in adult military members than in the civilian population [8].

Weight and BMI

This study showed a weak correlation between BMI and carpal tunnel size (cross-section area). Some studies have found a link between having a higher BMI and having more severe CTS, but the exact reason for the link is still unclear. However, CTS does not always improve when obese people lose weight, which suggests that causes of obesity may also cause CTS [9-19]. The repeated use of the wrist was blamed for this. Many computer operators and household workers have a higher incidence of CTS when compared to the general population [20-22]. Some researchers think that having more fat in the carpal tunnel may make the median nerve more likely to get pinched [23].

In a study by Mondelli et al., no correlation was found between BMI and CTS incidence in women and men. Further research is needed to determine whether the combination of carpal tunnel shape and higher BMI increases the risk for CTS [24].

We did not find any difference in carpal tunnel dimensions between nonpregnant and pregnant females in our study. Pregnancy is an associated risk factor for carpal tunnel syndrome. Though symptomatic females recover after delivery, pregnancy may not lead to changes in the dimensions of carpal tunnel. Compression of nerves in such cases may be associated with tissue edema [25]. Our findings are also supported by de Olivier et al. (2019), as they did not find any difference in fist size in the third trimester of pregnancy in females with and without CTS [26].

A potential limitation of this study is the posture of the wrist at the time of examination, which could affect the measurements. Even though most of the wrists were in a neutral position or bent no more than 10°, a slight variation in all measurements could be expected. Future studies should consider these limitations and try to reduce the differences. Another limitation is the low number of male participants, as participants were selected randomly based on inclusion and exclusion criteria, and no sexual priority was given.

Our study used a simple, inexpensive, and non-invasive approach for the study of carpal tunnels. The data collected in this way may be helpful in clinical practice and further research.

## Conclusions

Age, sex, weight, and BMI are predictors of the transverse diameter and cross-section area of the carpal tunnel. Wrist ratio was found to be similar in both sexes in this study. The effects of risk factors and the development of CTS were beyond the scope of our study. Wrist movements, injury to the wrist, occupation-related repeated trauma, cardiovascular conditions, and genetic composition contribute to the development of CTS. Our study plays a part in describing the morphologic differences between wrist and carpal tunnel size in male and female populations in India. More such studies need to be carried out to better understand the pathophysiology of CTS.
